# Correlation Analysis of Social Support, Family Resilience, and Family Function in Elderly Patients With Chronic Obstructive Pulmonary Disease: The Mediating Roles of Family Resilience

**DOI:** 10.1155/bmri/6640029

**Published:** 2026-03-09

**Authors:** Fang Yu, Xue Zhang, Yan Chang, Xiaona Zhang, Hongyan Lu

**Affiliations:** ^1^ Department of Neurology, General Hospital of Ningxia Medical University, Yinchuan, China, nxmu.edu.cn; ^2^ Department of Nursing, The First People’s Hospital of Yinchuan, Yinchuan, China, ycsyy.h.yynet.cn; ^3^ Department of Hematology, General Hospital of Ningxia Medical University, Yinchuan, China, nxmu.edu.cn; ^4^ Department of Nursing, General Hospital of Ningxia Medical University, Yinchuan, China, nxmu.edu.cn

**Keywords:** aged, family function, family resilience, mediation analysis, nursing, pulmonary disease chronic obstructive, social support

## Abstract

**Objective:**

This study analyzes the relationship between family resilience, social support, and family function of elderly patients with COPD. It explores the mediating effect of family resilience between social support and family function and the influence path among them, so as to provide evidence for medical staff to formulate targeted intervention measures and improve family function of elderly patients with COPD.

**Methods:**

Using a convenience sampling, 535 elderly patients with COPD were recruited in the respiratory and critical care departments of six tertiary hospitals in Ningxia, China from August 2022 to September 2023. The participants were assessed by general information questionnaire, family resilience assessment scale, social support assessment scale, family density, and adaptability scale. Pearson correlation analysis was used to evaluate the correlation between variables, and Model 4 in PROCESS 4.2 plugin was used to explore the mediating role of family resilience in the relationship between social support and family function.

**Results:**

Pearson correlation analysis showed that family function was positively correlated with social support (*r* = 0.406, *p* < 0.001) and family resilience (*r* = 0.622, *p* < 0.001), and family resilience was also positively correlated with social support (*r* = 0.361, *p* < 0.001). Mediation analysis revealed that social support was directly associated with family function (*β* = 0.556, 95*%*CI = 0.371, 0.741). Additionally, social support can also indirectly associated with family function via the mediation of family resilience (*β* = 0.526, 95*%*CI = 0.378, 0.672). The direct effect accounts for 51.39% of the total effect and the indirect effect accounts for 48.61% of the total effect.

**Conclusion:**

Family resilience plays a partial mediating role in social support and family function of elderly patients with COPD, and medical staff should pay attention to the integration of social support intervention and family resilience intervention to better improve family function of patients.

## 1. Introduction

COPD is a chronic respiratory disease characterized by persistent respiratory symptoms and limited airflow [1], which has the characteristics of high incidence, high disability rate, and high mortality rate [2], ranking the third cause of death in the world, second only to heart disease and stroke [3]. With the acceleration of the global population aging process, the elderly population has become the hardest hit area of COPD [4].

Studies have shown that the symptoms of elderly patients with COPD are complex, involving many aspects, such as physiology, psychology, society, etc., and often appear in the form of symptom groups. The symptoms in the groups cooperate and reinforce each other, which has a multiple negative impact on patients [5]. At the same time, the characteristics of COPD, which is difficult to recover and admitted to hospital repeatedly, bring pressure to the family in many aspects, such as care, economy, and social interaction, which leads to the decline of the overall function of the family [6].

Family function is the perception of family members on the overall function of the family, which can reflect the operation status, adaptability, and the relationship between family members to a certain extent. Studies have shown that the higher the level of family function, the more emotional support patients get, which is beneficial for enhancing patients’ confidence in overcoming diseases and promoting their treatment and rehabilitation [7].

Studies have shown [8] that social support services are associated with a reduced caregiving burden on families of patients with COPD, assist families in improving their ability to cope with the disease, and thus enhance the family function. In addition, with the development of positive psychology, family resilience has been proposed as a family advantage force, which is a force that helps families achieve good adjustment to maintain family stability [9]. Family resilience can improve family relations and help families cope with difficulties to a certain extent [10]. At the same time, some studies have shown that there is a certain correlation between family resilience and social support [11]. McCubbin’s theoretical model of family resilience also points out that family resources, support system, problem‐solving, and coping are interactive [12], so there may be some correlation between family resilience, social support, and family function of elderly patients with COPD.

At present, there are few studies to explore the relationship and mechanism among family resilience, social support, and family function. Therefore, this study analyzes the current situation of family function, social support, and family resilience of elderly patients with COPD, understands the correlation among them, and discusses the mediating role of family resilience in the relationship between social support and family function in elderly patients with COPD in order to provide basis for medical staff to formulate targeted intervention measures and improve family function of elderly patients with COPD.

## 2. Materials and Methods

### 2.1. Study Design and Participants

A cross‐sectional study was conducted in the Ningxia region of northwest China. Using convenient sampling method, elderly patients with COPD who were hospitalized in respiratory and critical care medicine departments of six tertiary hospitals in Ningxia from August 2022 to September 2023 were selected as the research object. The inclusion criteria for this study were as follows: (1) patients who meet the diagnostic criteria of the 2024 COPD Global Strategy, have stable condition, and those aged ≥ 60 years old [13], (2) who have clear awareness and can communicate and express themselves; (3) who participated in this survey voluntarily and signed an informed consent form. The exclusion criteria were as follows: (1) patients with major cardiovascular and cerebrovascular diseases, liver and kidney diseases, malignant tumors (lung cancer), and other end‐stage diseases; (2) patients who have suffered trauma events such as divorce and bereavement in recent 3 months [14–16].

### 2.2. Study Sample

The sample size was determined through a dual‐approach rationale to ensure both statistical power and model stability. First, an a priori power analysis was conducted using G∗Power 3.1 [17]. For the primary analysis, a multiple linear regression with two predictors (social support and family resilience) testing their association with family function. To achieve 80% power for detecting a small‐to‐medium effect size (f^2^ = 0.10) at a significance level of *α* = 0.05, a minimum of 123 participants was required. Second, considering that our hypothesized mediation model is optimally analyzed within a structural equation modeling (SEM) framework, we adhered to the sample size recommendations in this field that suggests a minimum sample size of 200 for stable SEM estimates, with samples above 500 being ideal for complex model testing and providing robust confidence intervals for indirect effects [18]. Integrating both criteria, we ultimately recruited 535 participants, to ensure the stability of model estimation.

### 2.3. Measurements

#### 2.3.1. Questionnaire of Patients’ General Information

The questionnaire was compiled by the researcher according to the research purpose and content, based on literature review, including demographic data and disease‐related data. Demographic data are mainly composed of age, education level, and medical payment method, etc. Disease‐related data are mainly composed of years of illness, the number of hospitalizations in the past year, the level of self‐care ability (the latest evaluation data of the patient’s nursing records), oxygen inhalation at home, and the use of ventilators.

#### 2.3.2. Family Adaptability and Cohesion Evaluation Scales II (FACES II)

The scale was compiled by Olson in 1979. This study used the Chinese version of FACESII‐CV revised in 1991 by Fei et al. [19], with two dimensions and 30 items, namely family intimacy and family adaptability. Likert’s five‐level scoring method was used, and 1~5 points were counted from “*not*” to “*always*”. The higher the score, the better the family function. Cronbach’s *α* of the scale is 0.68~0.85. This study used the scale to evaluate the family function of elderly patients with COPD, and Cronbach’s *α* was 0.90.

#### 2.3.3. Social Support Rating Scale (SSRS)

The scale was compiled by Xiao [20] in 1986, with three dimensions and 10 items, namely objective support, subjective support, and social support utilization. The score is according to the requirements of the item. Higher scores indicate more social support gained. The reliability coefficient of internal consistency of this scale is 0.92 and split‐half reliability is 0.89. This study used the scale to evaluate the social support of elderly patients with COPD, and Cronbach’s *α* was 0.75.

#### 2.3.4. Family Resilience Assessment Scale (FRAS)

The scale was compiled by Sixbey in 2005 according to the Walsh Family Resilience Framework. This study used the Chinese Family Resilience Assessment Scale revised in 2016 by Li et al. [21], with three dimensions and 32 items, namely family communication and problem‐solving, utilization of social resources, and maintaining a positive attitude. Likert’s four‐level scoring method was used, and the scores ranged from “*very different*” to “*very agree*” with 1–4 points. The higher the score, the higher the family resilience level. Cronbach’s *α* of the scale is 0.95. In this study, the scale was used to evaluate the family resilience level of elderly patients with COPD, and Cronbach’s *α* was 0.95.

### 2.4. Ethical Statement

All patients involved in this survey signed an informed consent form, and the study was approved by the Ethics Committee of General Hospital of Ningxia Medical University (2020‐643), complying with the declaration of Helsinki. Informed consent was obtained from the patients after the study’s aim was clarified. Confidentiality and anonymity were guaranteed by allocating a code number for each questionnaire. Patients were guaranteed that the data would be used only for research purposes. The right to withdraw from the study was confirmed.

### 2.5. Data Collection

With the permission and cooperation of hospitals and departments, the researchers selected 52 elderly patients with COPD who met the admission criteria for pre‐investigation, and made a preliminary analysis of the pre‐investigation data, thus making appropriate adjustments to some items of the general information questionnaire, and finally forming a formal version of the questionnaire. A formal investigation was conducted by the researcher and three investigators of the research team. The consent of the subjects before the investigation was obtained and the informed consent form was signed. Field investigation was adopted, and the subjects were asked to answer their own questions according to the actual situation. After the investigation, check whether there are any omissions in the questionnaire on the spot, and file the number after verification. Two researchers coded and inputted the data for statistical analysis, and a third person conducted spot checks on all the data. For individual missing values in the data, the mean of the variable would be used as a replacement.

### 2.6. Data Analysis

All statistical analyses were performed using IBM SPSS Statistic v21.0 software. Continuous variables with normal distribution were presented as the means and standard deviation (M ± SD), and the counting data was expressed by frequency and percentage. Pearson correlation analysis was used to analyze the correlation among family function, social support, and family resilience. The mediation effect was analyzed by Model 4 of process component of SPSS macro program. The Bootstrap method was used to set the confidence interval to 95% and the repeated sampling to 5000 times, and the mediation effect model was verified and analyzed. *p* < 0.05 was considered to indicate statistical significance.

## 3. Results

### 3.1. General Characteristics of the Participants

A total of 535 elderly patients with COPD were included in this survey, including 308 males and 227 females. The average age of the participants was 74.08 ± 7.97 years, and most of them had primary school education or below (75.3%). Farmers were the main occupation (63.7%). Most of the patients’ medical expenses were borne by the medical insurance for urban and rural residents (70.5%) and the medical insurance for urban workers (29.2%). The other characteristics of the participants were shown in Table 1.

**Table 1:** Characteristics of elderly patients with COPD (*N* = 535).

Variables	Categories	*n*	Percentage (%)
Gender	Male	308	57.6
	Female	227	42.4

Age (years)	60~69	146	27.3
	70~79	258	48.2
	80~89	121	22.6
	≥ 90	10	1.9

Educational level	Primary’s degree or below	403	75.3
	Junior high school	60	11.2
	Senior high school	61	11.4
	Bachelor’s degree or above	11	2.1

Marital status	Unmarried	5	0.9
	Married	420	78.5
	Divorced	2	0.4
	Widowed	108	20.2

Living style	Live alone	51	9.5
	Live with spouse	329	61.5
	Live with children	151	28.2
	Live with nanny	2	0.4
	Live in an old‐age care institution	2	0.4

Primary caregiver	Spouse	248	46.4
	Children	213	39.8
	Other relatives or friends	15	2.8
	Self‐care	59	11.0

Per capita monthly income (RMB)	< 1000	127	23.7
	1000~2999	220	41.1
	3000~4999	149	27.9
	≥ 5000	39	7.3

Type of health insurance	Self‐funded	2	0.4
	Medical insurance for urban and rural residents	377	70.5
	Medical insurance for urban employees	156	29.2

Course of disease (year)	< 5	222	41.5
	5~9	162	30.3
	10~14	114	21.3
	≥15	37	6.9

Combined with other chronic diseases	Yes	414	77.4
	No	121	22.6

Acute exacerbation in the past year (times)	0	255	47.7
	1	137	25.6
	2	76	14.2
	≥ 3	67	12.5
Inhale oxygen or use a ventilator at home (hours)	0	309	57.8
	< 12	195	36.4
	≥ 12	31	5.8

Self‐care ability of daily life	Self‐care completely	14	2.6
	Mild dependence	391	73.1
	Moderate dependence	111	20.7
	Heavy dependence	19	3.6

### 3.2. The Correlation Between Family Function, Social Support, and Family Resilience

The scores of family function, social support, and family resilience of elderly patients with COPD were 94.07 ± 12.88, 38.96 ± 4.83, and 98.40 ± 11.53, respectively. The result of Pearson correlation analysis showed that family function was positively correlated with social support (*r* = 0.406, *p* < 0.001), family function was positively correlated with family resilience (*r* = 0.622, *p* < 0.001), and family resilience was positively correlated with social support (*r* = 0.361, *p* < 0.001) as shown in Table 2.

**Table 2:** The correlation analysis of family resilience, social support, and family function in elderly patients with COPD (*N* = 535).

Variables	Family resilience	Family function	Social support	*M* ± *S* *D*
Family resilience	1.000 ^∗∗^			98.40 ± 11.53
Family function	0.622 ^∗∗^	1.000 ^∗∗^		94.07 ± 12.88
Social support	0.361 ^∗∗^	0.406 ^∗∗^	1.000 ^∗∗^	38.96 ± 4.83

*Note:* M: mean, SD: standard deviation.

^∗∗^ *p* < 0.001.

### 3.3. The Mediating Effect of Family Resilience on Social Support and Family Function in Elderly Patients With COPD

Multiple level regression analysis was conducted with family function as the dependent variable, social support as the independent variable, and family resilience as the mediating variable. The results showed a moderate positive correlation between social support and family function, and social support can explain 16.5% of the variation in family function (*β* = 0.406, *p* < 0.001, *R* = 0.406, *R* ^2^ = 0.165). There was a moderate positive correlation between social support and family resilience, and social support can explain 13.0% of the variation in family resilience (*β* = 0.361, *p* < 0.001, *R* = 0.361, *R* ^2^ = 0.130). Both variables of family resilience and social support were strongly positively correlated with family function, and together explained 42.5% of the variation in family function (*β* = 0.547, *p* < 0.001, *R* = 0.652, *R* ^2^ = 0.425). This indicated that the explanatory power of the model significantly improves after the inclusion of family resilience as shown in Table 3.

**Table 3:** The regression analysis of the family resilience mediation model (*N* = 535).

Dependent variable	Independent variable	*R*	*R* ^2^	*F*	*β*	*t*
Family function	Social support	0.406	0.165	104.964 ^∗∗^	0.406	10.245 ^∗∗^
Family resilience	Social support	0.361	0.130	79.655 ^∗∗^	0.361	8.925 ^∗∗^
Family function	Social support	0.406	0.165	104.964 ^∗∗^	0.208	5.912 ^∗∗^
	Family resilience	0.652	0.425	196.417 ^∗∗^	0.547	15.514 ^∗∗^

^∗∗^ *p* < 0.001.

**Figure 1:**
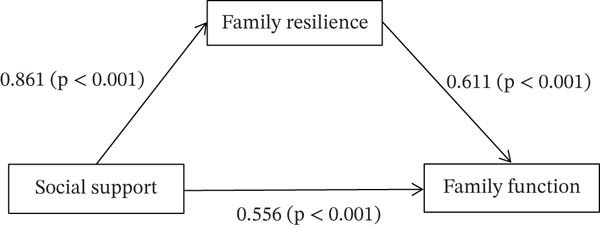
The mediating effect of family resilience in the relationship between social support and family function in elderly patients with COPD.

Using Model 4 of SPSS plug‐in PROCESS provided by Hayes to analyze the mediating effect of family resilience on social support and family function in elderly patients with COPD, with social support as the independent variable, family function as the dependent variable and family resilience as the mediating variable. The result of path coefficient is shown in Figure 1.

The mediating effect was tested by Bootstrap analysis. The results showed that social support was directly associated with family function (*β* = 0.556, 95*%*CI = 0.371, 0.741). In addition, social support also indirectly associated with family function via the mediation of family resilience (*β* = 0.526, 95*%*CI = 0.378, 0.672). It showed that family resilience plays a partial mediating effect between social support and family function, with direct effect accounting for 51.39% and indirect effect accounting for 48.61%, as shown in Table 4.

**Table 4:** Analysis of the mediating effect of family resilience between social support and family functioning (*N* = 535).

Effect relation	Influence path	Effect value	SE	LLCI	ULCI	Effect quantity
Indirect effect	Social support→Family resilience→Family function	0.526	0.076	0.378	0.672	48.61%
Direct effect	Social support→Family function	0.556	0.094	0.371	0.741	51.39%
Total effect	Social support→Family function	1.082	0.106	0.874	1.289	

## 4. Discussion

### 4.1. Analysis of the Status of Family Function in Elderly Patients With COPD

The results of this study showed that the family function of elderly patients with COPD is at a moderate‐to‐low level, which is consistent with the research findings of Tian et al. [22]. An analysis of the reasons, first of all, 75.3% of the subjects in this study have primary school education or below, and their education level is generally low. Patients with low education level have weak learning ability for disease knowledge, which is not conducive to solving bad family events; thus, the family function is worse. Secondly, in this study, 64.8% of patients have a family income of less than 3000 RMB per month, and 70.5% have medical insurance for urban and rural residents. The overall economic situation of patients is general, while COPD is recurrent and progressive. The expenses and time for disease control and rehabilitation are very large, and the family’s coping ability is limited, which leads to poor communication among family members due to money, thus destroying the family atmosphere and affecting family functions. In addition, in this study, 61.5% patients live with their spouses, and 46.4% patients’ main caregivers are spouses. Studies have shown that [23] children accompany the elderly rarely because of work or other affairs. When the elderly encounter difficulties, they are used to bearing silently or relying on their spouses, less actively seeking material and emotional support from their children, less communication between family members, and it is often difficult to cope with major and unexpected events, which reduces family functions. Therefore, the government and medical institutions should strengthen the propaganda of COPD‐related knowledge, improve disease awareness, carry out various activities and encourage family members to participate, so as to enhance the emotional communication and disease prevention ability among family members and improve family functions. In addition, medical staff should evaluate the family function of elderly patients with COPD in a timely and effective manner, understand the operation status of their family system, and guide family members actively and effectively if they find family dysfunction, so as to help them solve family problems and better improve family function.

### 4.2. The Correlation Analysis of Family Function, Social Support, and Family Resilience in Elderly Patients With COPD

The results of this study showed that the family function of elderly patients with COPD is positively correlated with social support. Social support is the information sharing, material assistance, psychological support, and value transmission brought by interpersonal communication, and it is also an important resource for families to buffer stress. Moderate social support affects the adaptability of families to stress events directly or indirectly, thus affecting family function [24]. Correlation analysis showed that social support and family resilience of elderly patients with COPD are also positively correlated, that is, the more social support patients receive, the higher their family resilience level, which is consistent with previous research results [25]. Donnellan and others think that social support is more related to family resilience than family support [26]. However, because of the symptoms of dyspnea, decreased activity endurance, and other factors, the scope and frequency of the participation of the elderly patients with COPD in social activities are greatly limited. Over time, patients are unwilling to participate in social group activities or communicate with outsiders, and the role of the families′ social support for elderly patients with COPD is gradually weakened, which in turn affects family resilience. Besides, this study showed that there is a significant positive correlation between family resilience and family function in elderly patients with COPD. Patterson believes that when faced with difficulties, the higher level of family function, the stronger the family’s ability to cope with crises [27]. The reason may be that the higher level of family function, the better the relationship between family members, the more conducive to family communication and family harmony and stability, and thus enhance the family’s ability to cope with the crisis. Moreover, good family relations contribute to family harmony and communication, and enhance the family’s ability to resist adversity. This suggests that in their daily work, medical staff should not only pay attention to patients’ physical and mental health, but also evaluate their whole family system, evaluate their family function and fully tap their family resources to help patients’ families cope with the crisis successfully and realize positive adaptation.

### 4.3. The Analysis of the Mediating Effect of Family Resilience on the Relationship Between Family Function and Social Support in Elderly Patients With COPD

Mediating effect analysis showed that family resilience plays a partial mediating role in the relationship between family function and social support in elderly patients with COPD. Social support was not only directly associated with family function, but also indirectly associated with family function through family resilience, accounting for 48.61% of the total effect. The reason may be that the higher the level of social support, the more family members can use internal and external resources to collect medical information, find social resources actively to expand the support network, and establish a cooperative relationship with medical staff to adapt to the family crisis. Seeking social support system in stressful times can strengthen the ability of family communication and problem solving, promote the cohesion and flexibility of family members and their relatives, stimulate patients to have a positive development track, and help patients cope with diseases more optimistically and tenaciously [28]. Families with high level of family resilience express their feelings and share clear responsibilities, which can make effective decisions to solve problems in an emergency and help patients get more family support and protection, thus improving their family function. Therefore, while expanding patients’ social support network, clinical medical staff can integrate family resilience‐oriented intervention measures into social support interventions to help the families of elderly patients with COPD tap their internal advantages and strengths actively, so as to give full play to the important role of family resilience in social support and promote family function, and improve the clinical intervention effect.

## 5. Conclusion

The family function of elderly patients with COPD is at a moderate‐to‐low level, and family resilience plays a partial mediating role in the relationship between social support and family function. Therefore, social support intervention and family resilience intervention can be integrated in clinic to expand the social support obtained by elderly patients with COPD, improve their family resilience level, to help patients create a good living environment, and then promote the improvement of their family function.

Since this study is a cross‐sectional study, it is impossible to infer the causal relationship among social support, family resilience, and family function. In the future, it is necessary to further consider the longitudinal study to explore the correlation between these variables and explore the potential mechanism of patient interaction between different stages of the disease. In addition, the participants in this study are all from the northwest region of China, which may limit the generalizability of the research results to other cultural or geographical backgrounds. Future research needs to be conducted in a broader population to validate our findings.

## Funding

This study was supported by Ningxia Science and Technology Benefit People Special Project, China, 2023CMG03047; No.394 [2023] of General Hospital of Ningxia Medical University, 2023 New Master’s Talents Training Program, 394 [2023].

## Conflicts of Interest

The authors declare no conflicts of interest.

## Data Availability

The datasets generated during and/or analyzed during the current study are available from the corresponding author upon reasonable request.
